# Medical Schools of Canada

**Published:** 1861-05

**Authors:** 


MEDICAL SCHOOLS OF CANADA.—The number of students in attendance at the medical schools of Canada for 1860-61 are as follows:—

| | |
|---|---|
| University of McGill College, Montreal | 124 |
| Toronto School of Medicine | 81 |
| Montreal School of Medicine | 43 |
| University of Laval, Quebec | 32 |

